# Effect of repetitive transcranial magnetic stimulation modulating supplementary motor area in stroke rehabilitation: Systematic review of randomized controlled trials

**DOI:** 10.3934/Neuroscience.2026003

**Published:** 2026-01-28

**Authors:** Ayesha Juhi, Shreya Sharma, Dinesh Bhatia, Suman Dhaka, Rajesh Kumar, Deepak Kumar, Rintu Kumar Gayen, Himel Mondal

**Affiliations:** 1 Department of Physiology, All India Institute of Medical Sciences, Deoghar, Jharkhand, India; 2 Neuromodulation Laboratory, Department of Physiology, All India Institute of Medical Sciences, Deoghar, Jharkhand, India; 3 Department of Biomedical Engineering, North-Eastern Hill University, Shillong, Meghalaya, India; 4 School of Liberal Arts and Center for Brain Science and Application, Indian Institute of Technology, Jodhpur, India; 5 Department of Internal Medicine, All India Institute of Medical Sciences, Deoghar, Jharkhand, India; 6 Department of Physical Medicine and Rehabilitation, All India Institute of Medical Sciences, Deoghar, Jharkhand, India; 7 Electronics and Communication Engineering, Institute of Engineering and Management, Kolkata, India

**Keywords:** stroke rehabilitation, supplementary motor area, repetitive transcranial magnetic stimulation, functional connectivity, balance, motor recovery

## Abstract

**Background:**

Repetitive transcranial magnetic stimulation (rTMS) is a promising adjunct in stroke rehabilitation, with increasing focus on the supplementary motor area (SMA) for its role in motor and language network reorganization. This systematic review evaluated randomized controlled trials (RCTs) assessing rTMS targeting the SMA or modulating its connectivity to improve motor and language outcomes in adults after ischemic or hemorrhagic stroke.

**Methods:**

A comprehensive literature search was performed across PubMed, Web of Science, and Google Scholar, focusing on peer-reviewed RCTs published in English. Studies were eligible if they involved adults with stroke at any recovery stage and assessed rTMS targeting or analyzing the SMA. Two independent reviewers screened records, extracted data, and assessed risk of bias using the Cochrane Risk of Bias 2.0 tool. The review adhered to PRISMA guidelines and was registered in PROSPERO (CRD420251051684).

**Results:**

Among 74 screened for full text, five RCTs were included. Across studies, rTMS interventions targeting the SMA or modulating SMA-related networks improved motor and language outcomes in stroke patients. Neuroimaging measures consistently demonstrated increased SMA activation and strengthened SMA-primary motor cortex (M1) connectivity after rTMS, which correlated with clinical improvements. Balance and postural control outcomes showed the most consistent benefits, while upper-limb motor and language improvements varied in magnitude and follow-up duration.

**Conclusion:**

Heterogeneity in studies, small sample sizes, and concerns about randomization and reporting limit our ability to draw definitive conclusions. SMA-targeted or network-level rTMS appears to facilitate functional recovery after stroke, supported by neuroimaging evidence of brain network reorganization. Further large, standardized RCTs are necessary to establish optimal protocols, confirm efficacy, and assess long-term outcomes.

## 1. Introduction

Stroke is a leading cause of long‑term disability worldwide, and motor impairments are among its most disabling consequences [1],[2]. Despite improvements in acute care and rehabilitation, many survivors continue to have persistent deficits such as impairments in balance, gait, and movement that limit their function and quality of life [3],[4]. Neuromodulation has emerged as a promising adjunct to rehabilitation, intended to boost neuroplasticity and foster reorganization of cortical networks [5],[6].

The supplementary motor area (SMA), situated on the medial frontal cortex, contributes importantly to motor planning, initiation, and coordination of complex actions and participates in interhemispheric motor network interactions that can support compensatory reorganization after stroke [1],[7]. Applying neuromodulatory approaches, including repetitive transcranial magnetic stimulation (rTMS), transcranial direct current stimulation (tDCS), and invasive stimulation to the SMA, is gaining scientific interest, although most clinical work to date has focused on the primary motor cortex (M1). The evidence for SMA-targeted interventions remains limited, with studies often constrained by small sample sizes and heterogeneous outcome measures [8],[9].

Given the SMA's role in higher-order motor control and recovery potential, a systematic synthesis of SMA-targeted neuromodulation in stroke is warranted. Hence, this review assesses stimulation techniques and protocols, along with patient populations and measured outcomes, in order to evaluate the therapeutic worth of SMA-targeted neuromodulation, while providing directions for future research and clinical practice in stroke recovery programs.

## 2. Materials and methods

### 2.1. Protocol registration

This systematic review was prospectively registered in the International Prospective Register of Systematic Reviews (PROSPERO) under registration number CRD420251051684. The review was conducted in accordance with the Preferred Reporting Items for Systematic Reviews and Meta- Analyses (PRISMA) guidelines to ensure transparency and methodological rigor throughout the study process.

### 2.2. Search strategy

A comprehensive literature search was conducted on May 20, 2025, by two authors across three major electronic databases: PubMed, Web of Science (WOS), and Google Scholar (up to the first 10 pages, 100 search results). A consensus was reached to finalize the number of articles per database. The objective was to identify studies that investigated the use of rTMS in stroke rehabilitation, with specific attention to interventions targeting the supplementary motor area SMA. The search strategy employed a combination of relevant keywords and Boolean operators to maximize the retrieval of pertinent studies. The terms used included *stroke*, *stroke rehabilitation*, *repetitive transcranial magnetic stimulation*, and *supplementary motor area*. The detailed keywords for the search are available in Supplementary Table 1.

### 2.3. Inclusion and exclusion criteria

Eligible studies included those that evaluated adults with ischemic or hemorrhagic stroke at any stage of recovery and investigated rTMS targeting the SMA or analyzed SMA-related effects. Only randomized controlled trials published in peer-reviewed journals in English were included. Studies available up to the search date (May 20, 2025) were included. Studies were excluded if they did not involve stroke patients, did not use rTMS, did not include the SMA as a stimulation target or analysis region, or were animal studies, reviews, protocols, editorials, or conference abstracts.

### 2.4. Screening and selection

Two reviewers independently screened titles and abstracts, followed by a full-text review of potentially eligible studies. Disagreements were resolved through discussion, with arbitration by a third author when necessary.

### 2.5. Data extraction and risk of bias assessment

Data were extracted independently by two reviewers using a standardized form, covering study design, participant characteristics, intervention parameters, stimulation site, outcomes, results, and adverse events. Risk of bias for all included studies was assessed using validated tools specific to each study design. The Cochrane Risk of Bias 2.0 (RoB 2) tool was applied for evaluating five domains, including randomization, deviations from intended interventions, missing outcome data, measurement of outcomes, and selective reporting. The rating was performed based on the study characteristics and categorized into three levels: low risk, some concerns, and high risk. The credibility of the screening and data extraction process and inter-rater reliability were assessed between the two independent reviewers. The level of agreement was quantified using Cohen's Kappa statistic, with a value of ≥0.6 considered acceptable for methodological credibility. After the analysis of Cohen's Kappa, a meeting was conducted among the raters to finalize the risk of bias after consensus.

**Table 1.** Studies and their characteristics.

Author/year	Study design	Country	Population (P in PICO)	Sample size in the intervention group	Intervention details (I in PICO: dose, frequency, duration, type)	Sample size in the control group	Control details (C in PICO: dose, frequency, duration, type)	Outcome measures (O in PICO: specify the tools/scales used)	Summary result
Zhao et al., 2024 [10]	Randomized control-led trial	China	Adults with subcortical ischemic stroke (first onset, 2–12 weeks), motor dysfunction (FMA-UE < 32, FMA-LE < 18, BBS < 20), MMSE > 21	15 (SMA group), 16 (M1 group)	SMA group 10 Hz rTMS to SMA, 100% RMT, 2.5 s trains, 10 s intertrain interval, 2400 pulses/session 20 min/day, 5 days/week 4 weeks	15	Sham rTMS	FMA-UE, FMA-LE, Berg Balance Scale (BBS), fMRI measures (ALFF, ReHO, FC)	10 Hz SMA-rTMS (4 weeks) improved motor and balance outcomes. SMA stimulation produced greater balance gains vs. M1 and sham, with correlated Fmri changes
Gan et al., 2024 [11]	Randomized control-led trial	China	Adults with subacute stroke (1–6 months), Broca's aphasia, first onset, right-handed, ≥6 years of education	9	Low-frequency (1 Hz) rTMS to the right hemisphere, 20 min/day, 5 days/week × 4 weeks, followed by 30 min/day speech and language therapy	9	Sham rTMS + speech and language therapy	Western Aphasia Battery Revised (WAB-R), Stroke and Aphasia Quality of Life Scale-39 (SAQOL-39), Non-Language-Based Cognitive Assessment (NLCA), fNIRS	1 Hz right-hemisphere rTMS + speech therapy improved Broca aphasia naming with sustained 3-month effects and task-related fNIRS activation decreases, indicating functional reorganization
Xia et al., 2022 [12]	Randomized controlled trial	China	Adults aged 18–80 years with unilateral subacute or chronic ischemic/hemorrhagic stroke (>3 weeks), motor dysfunction (FMA-LE < 34), balance dysfunction (BBS < 56), able to stand alone ≥5 min	11 (CB-M1 group), 10 (CB-SMA group)	iTBS to CB-M1 or CB-SMA; 3 pulses at 50 Hz repeated every 200 ms (5 Hz), 2 s trains every 10 s, total 600 pulses; single session	10	iTBS to unilateral cerebellum only (CB-single), same parameters	COP parameters (COP speed, acceleration, ML-COPd, AP-COPd, etc.), fNIRS for functional connectivity	Cerebellum–cerebrum paired iTBS (single session) showed target-dependent FC changes; CB–SMA pairing produced the strongest inhibitory/FC effects on motor network in this pilot fNIRS study
Guo et al., 2021 [13]	Randomized controlled trial	China	33	HF: 11; LF: 12	HF: 10 Hz over ipsilesional M1, 90% RMT, 1500 pulses/session (30 × 50), 10 consecutive days; LF: 1 Hz over contralesional M1, 90% RMT, 900 pulses/session (30 × 30), 10 days	Sham-10	Sham rTMS delivered with the same parameters as LF but without induced current (placebo)	Fugl-Meyer Assessment (FMA), Barthel Index (BI), NIHSS; neuroimaging: resting-state fMRI (ICA and seed-based intramotor FC among bilateral M1, SMA, PMA); Pearson correlations between FC and clinical change	It compares HF vs. LF rTMS effects on motor-network reorganization and motor recovery: both real rTMS improved outcomes vs. sham, with HF producing greater ipsilesional FC increases correlated with motor gains
Li et al., 2016 [14]	Randomized controlled trial	China	Patients with unilateral subcortical ischemic stroke (MCA territory), <1 week onset, right-handed, no prior stroke	7	rTMS to ipsilesional M1, 50 trains × 20 pulses, 5 Hz 120% RMT of unaffected extremity, 10 consecutive days starting 5 days post-stroke	5	Sham rTMS	NIHSS, Barthel Index (BI), Fugl-Meyer Assessment (FMA)	rTMS (7 patients) produced significant motor improvement (NIHSS ↓ FMA and BI ↑) after 10 days of 5 Hz ipsilesional stimulation, and rs-fMRI showed increased FC between ipsilesional M1 and contralesional M1, SMA, bilateral thalamus and postcentral gyrus with decreased ipsilesional frontal–parietal FC; high-frequency ipsilesional rTMS appears to facilitate motor recovery through functional reorganization and is a promising, safe rehabilitation strategy for early subcortical ischemic stroke patients

Note: SMA, supplementary motor area; M1, primary motor cortex; HF, high frequency; LF, Low Frequency; rTMS, repetitive transcranial magnetic stimulation; RMT, resting motor threshold; iTBS, intermittent theta burst stimulation; CB, cerebellum; FMA, Fugl-Meyer Assessment; FMA-UE, Fugl-Meyer Assessment—upper extremity; FMA-LE, Fugl-Meyer Assessment—lower extremity; BBS, Berg Balance Scale; MMSE, mini-mental state examination; fMRI, functional magnetic resonance imaging; ALFF, amplitude of low-frequency fluctuation; ReHo, regional homogeneity; FC, functional connectivity; WAB-R, Western Aphasia Battery—revised; SAQOL-39, Stroke and Aphasia Quality of Life Scale—39; NLCA, Non-Language-Based Cognitive Assessment; fNIRS, functional near-infrared spectroscopy; COP, center of pressure; ML-COPd, mediolateral center of pressure displacement; AP-COPd, anteroposterior center of pressure displacement; BI, Barthel Index; NIHSS, National Institutes of Health Stroke Scale; ICA, independent component analysis; PMA, premotor area; MCA, middle cerebral artery; rs-fMRI, resting-state functional magnetic resonance imaging. PICO represents population, intervention, control, and outcome. In the tables, “↑” indicates an increase and “↓” indicates a decrease in the reported measures.

## 3. Results

**Figure 1.**
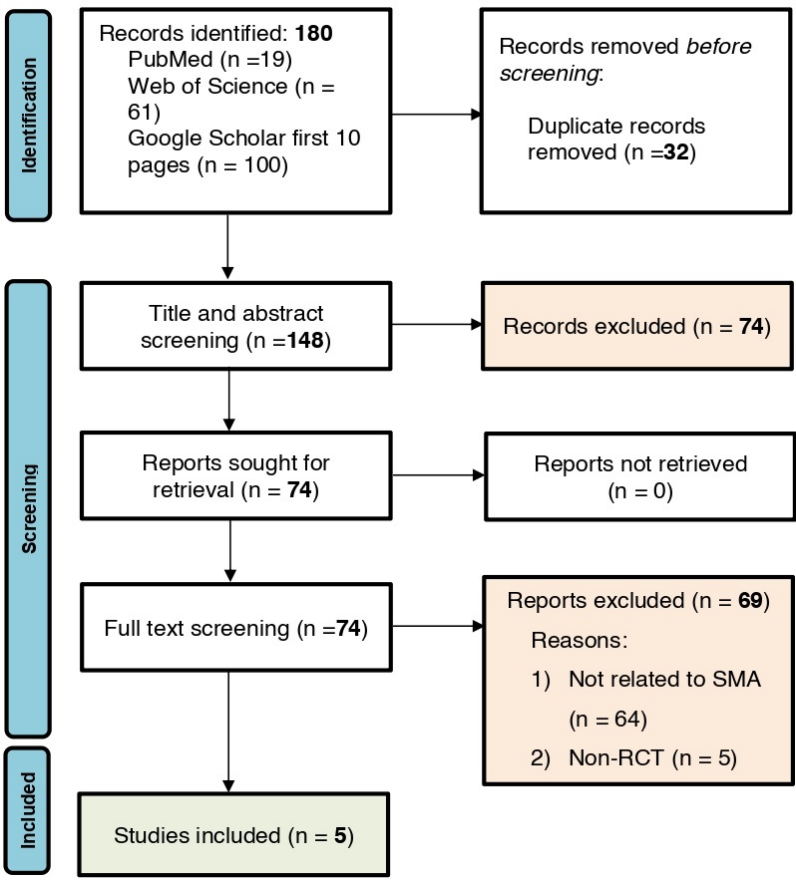
PRISMA flowchart showing the number of studies in various stages.

A total of 180 records were identified through PubMed (n = 19), Web of Science (n = 61), and Google Scholar (n = 100). After removing 32 duplicates, 148 records underwent title and abstract screening, with 74 records excluded. All 74 full-text articles were retrieved for assessment, and 69 were excluded due to either lacking SMA targeting or network-level SMA analysis (n = 64) or not being randomized controlled trials (n = 5). Ultimately, 5 studies were included in the systematic review (Figure 1).

The reviewed studies (Table 1) [10]–[14] demonstrated beneficial effects of rTMS on motor and language outcomes in stroke rehabilitation. Zhao et al. (2024) showed that 10 Hz rTMS targeting the supplementary motor area (SMA) over 4 weeks significantly improved motor function and balance compared to sham and M1 stimulation, accompanied by fMRI changes [10]. Gan et al. (2024) reported that low-frequency (1 Hz) rTMS over the right hemisphere combined with speech therapy improved naming in Broca's aphasia patients, with sustained benefits at 3 months and task-related fNIRS activation reductions indicating functional reorganization [11]. Xia et al. (2022) found that one paired iTBS to cerebellum–SMA induced greater functional connectivity modulation than cerebellum alone, suggesting enhanced motor network effects in stroke patients [12]. Guo et al. (2021) demonstrated that both high-frequency (10 Hz) and low-frequency (1 Hz) rTMS improved motor outcomes compared to sham, with high-frequency rTMS showing stronger increases in ipsilesional motor network connectivity, correlating with better recovery [13]. Li et al. (2016) showed that early high-frequency ipsilesional rTMS (5 Hz) significantly improved motor scores and functional connectivity between ipsilesional and contralesional motor areas [14].

**Figure 2.**
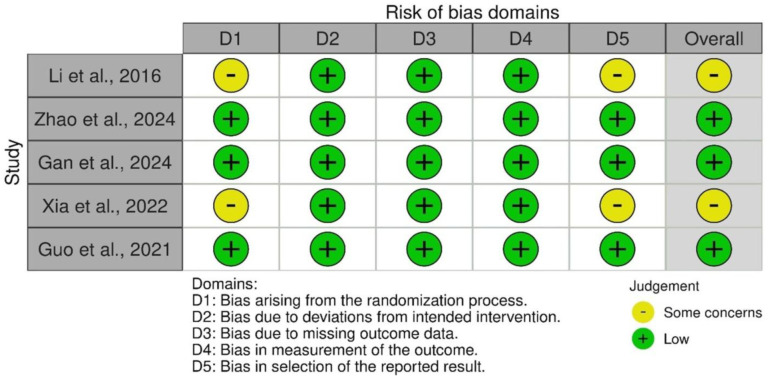
Risk of bias of the studies.

The risk of bias is shown in Figure 2. Most studies showed a low risk of bias across all domains. Li et al. (2016) and Xia et al. (2022) had some concerns regarding bias in the randomization process and selection of the reported results [12],[14]. Overall, 3 out of 5 studies were judged to have a low risk of bias.

## 4. Discussion

The overall findings of this systematic review support the growing evidence that rTMS is a promising adjunctive intervention in stroke rehabilitation. The observed improvements in motor and language functions suggest that rTMS facilitates neuroplasticity by modulating cortical excitability and enhancing functional connectivity within relevant neural networks [15],[16]. Importantly, studies combining rTMS with conventional therapies, such as speech therapy or physical rehabilitation, appear to produce more robust and sustained functional gains, highlighting the synergistic potential of multimodal interventions [17].

The supplementary motor area acts as a key hub within the motor and language networks, facilitating interhemispheric communication and compensatory activation after stroke [18]. High-frequency rTMS applied to the SMA likely enhances cortical excitability and strengthens connectivity with regions such as the primary motor cortex, premotor cortex, and language-related areas, thereby supporting recovery [19]. Compared with other neuromodulation approaches such as transcranial direct current stimulation (tDCS) and transcranial alternating current stimulation (tACS), rTMS provides greater focality and depth of stimulation, enabling more precise modulation of targeted cortical circuits [20].

The consistent use of neuroimaging tools, including fMRI and fNIRS, across the studies underscores the role of functional reorganization in mediating clinical improvements. These findings indicate that rTMS not only influences behavioral outcomes but also promotes measurable changes in brain network dynamics, supporting its mechanistic rationale [10],[21],[22]. However, variations in stimulation parameters, target regions, and intervention timing reflect a lack of standardization, which complicates direct comparisons and calls for future protocol harmonization.

Evidence increasingly supports the view that the SMA plays a critical role in stroke recovery through its network-level interactions, even when not directly targeted by stimulation. Several studies have shown that stimulating regions such as M1 or the right inferior frontal gyrus (IFG) indirectly modulates SMA activity and strengthens SMA-M1 connectivity, which often correlates with functional improvements in motor and language outcomes [13],[14],[16]. In motor recovery cohorts, both resting-state and task-based neuroimaging measures consistently demonstrated enhanced connectivity between SMA and primary motor regions, closely tracking improvements in clinical assessments like the Fugl-Meyer Assessment, Action Research Arm Test (ARAT), and gait performance [15]–[17],[23].

Similarly, in patients with post-stroke aphasia, low-frequency rTMS to the right IFG combined with therapy led to shifts in SMA activation toward more efficient patterns, as measured by fNIRS and fMRI, coinciding with improvements in naming and speech production [10],[24]. These findings emphasize the role of the SMA as a compensatory hub, supporting recovery even when not directly stimulated. Theoretical frameworks suggest that dysfunctional SMA inhibition over M1 contributes to motor deficits, which aligns conceptually with the network model of post-stroke dysfunction, though direct evidence supporting SMA as a primary target remains limited [25].

Among clinical outcomes, balance and postural control showed the most reliable signs of SMA engagement, where interventions targeting the SMA consistently outperformed or matched M1 stimulation in head-to-head comparisons [15]. Upper-limb motor outcomes improved either through direct SMA stimulation or via enhanced SMA connectivity following M1 stimulation, though the magnitude and consistency of effects varied depending on assessment tools and follow-up durations [16],[21],[23]. For gait outcomes, single-session SMA stimulation did not yield significant changes, likely due to underdosing and the chronic stage of stroke, rather than indicating target irrelevance [13],[16],[26].

A study by Jiang et al. reported the effects of 10 Hz rTMS over the supplementary motor area on balance and postural control in stroke patients. The rTMS group showed significantly greater improvements in balance and postural stability compared to the sham group. These findings suggest that rTMS targeting the supplementary motor area may serve as an effective adjunct therapy for enhancing postural recovery after stroke [27].

Language studies reflected a parallel pattern: stimulation of traditional language regions reshaped SMA activity, with this reorganization accompanying clinical improvements. This supports the concept of the SMA as a network amplifier that facilitates practice-dependent gains across motor and language domains. Multimodal imaging consistently converged on an SMA-centered mechanism, showing increased SMA activation and SMA-M1 coupling following effective stimulation and rehabilitation, which correlated with clinical benefits [17].

Moreover, emerging work on individual differences suggests that baseline network characteristics, including SMA connectivity, may predict responsiveness to interventions such as intermittent theta burst stimulation (iTBS). This reinforces the need for network-informed approaches to targeting and dosing, moving beyond a region-centric view toward a more integrative model of stroke rehabilitation.

If balance or postural stability in subacute stroke is the goal, SMA is a practical initial target. High-frequency protocols, multi-session dosing, and combination with task-specific balance training should be considered. For upper-limb recovery, SMA targeting is sensible but with conflicting results; therefore, baseline SMA connectivity and lesion location stratification may improve responder selection. In aphasia, even with IFG stimulation, SMA activation should be monitored as an effective network-recruitment biomarker, and intensive language therapy should be incorporated to capitalize on plasticity.

Heterogeneity among the included studies primarily arose from variations in study design, participant characteristics, and stimulation parameters. The studies differed in stroke phase (acute, subacute, and chronic), lesion location (motor vs. language networks), and functional domains assessed (motor, balance, or language recovery). Substantial variability was also observed in rTMS protocols, including stimulation frequency (1, 5, and 10 Hz iTBS), target regions (SMA, M1, cerebellum, or inter-hemispheric sites), pulse number, intensity, and session duration. Differences in outcome measures, ranging from clinical scales to neuroimaging and neurophysiological indices, further contributed to methodological and statistical heterogeneity across trials [10]–[14].

The strength of this systematic review lies in its comprehensive synthesis of recent high-quality randomized controlled trials investigating the role of SMA-targeted and network-level rTMS in stroke rehabilitation, supported by objective neuroimaging measures such as fMRI and fNIRS. By focusing on both motor and language outcomes, the review highlights the broader applicability of SMA network modulation across functional domains, emphasizing mechanistic insights alongside clinical effects.

### 4.1. Limitations

The relatively small number of included trials and heterogeneity limit the ability to draw firm conclusions or perform a quantitative meta-analysis. The literature search was conducted using only three electronic databases (PubMed, Web of Science, and Google Scholar). While these databases cover a broad range of biomedical and scientific literature, restricting the search to these sources may have led to the exclusion of relevant studies indexed in other databases such as Embase and Scopus. The Google Scholar search was limited to the first 10 pages (100 results) to maintain feasibility and focus on the most relevant studies, though this approach may have introduced selection bias. Gray literature sources such as conference proceedings, theses, and trial registries were not included, which might have influenced the comprehensiveness of the review. This may introduce selection bias and limit the comprehensiveness of the review.

### 4.2. Future scope

Future research should aim to validate these findings through large, multicenter randomized controlled trials using standardized rTMS protocols targeting the supplementary motor area. Incorporating advanced neuroimaging and electrophysiological techniques may help clarify the underlying mechanisms of SMA-mediated recovery. Long-term follow-up studies are warranted to assess the persistence of therapeutic effects. Additionally, individualized stimulation parameters and integration of rTMS with task-specific rehabilitation could enhance functional outcomes and support the translation of SMA-targeted neuromodulation into routine stroke care.

## 5. Conclusions

The limited number of available trials, combined with small samples and heterogeneous stimulation parameters, restricts the certainty of the current evidence. Although some studies suggest that SMA-targeted or network-level rTMS may support improvements in motor, balance, and language outcomes, these effects remain preliminary and are not consistently demonstrated. Neuroimaging findings indicating increased SMA activation and stronger connectivity with M1 offer supportive but still exploratory insights into potential mechanisms. Overall, larger and methodologically robust randomized controlled trials with standardized protocols are needed before firm conclusions can be drawn regarding efficacy, optimal parameters, and long-term outcomes.

## Use of AI tools declaration

ChatGPT-4o (OpenAI) was used to edit the language and grammar of the article. The content was checked thoroughly after editing by the chatbot, and the authors take full responsibility for the content.


