# Supplementary material for: Effect of repetitive transcranial magnetic stimulation modulating supplementary motor area in stroke rehabilitation: Systematic review of randomized controlled trials
Source: AIMS Neurosci. 2026 Jan 28;13(1):50–63. doi: 10.3934/Neuroscience.2026003 (PMC13086594; doi:10.3934/Neuroscience.2026003)
Supplement: Supplementary file 1 [file neurosci-13-01-003-s001.pdf]

Neuroscience

https://www.aimspress.com/journal/neuroscience

AIMS Neuroscience, 13(1): 50–63.
DOI: 10.3934/Neuroscience.2026003
Received: 25 September 2025
Revised: 01 December 2025
Accepted: 06 January 2026
Published: 28 January 2026

Review

Effect of repetitive transcranial magnetic stimulation modulating supplementary motor area in stroke rehabilitation: Systematic review of randomized controlled trials

Ayesha Juhi[1], Shreya Sharma[2,*], Dinesh Bhatia[3], Suman Dhaka[4], Rajesh Kumar[5], Deepak Kumar[6], Rintu Kumar Gayen[7] and Himel Mondal[1,*]

1 Department of Physiology, All India Institute of Medical Sciences, Deoghar, Jharkhand, India
2 Neuromodulation Laboratory, Department of Physiology, All India Institute of Medical Sciences, Deoghar, Jharkhand, India
3 Department of Biomedical Engineering, North-Eastern Hill University, Shillong, Meghalaya, India
4 School of Liberal Arts and Center for Brain Science and Application, Indian Institute of Technology, Jodhpur, India
5 Department of Internal Medicine, All India Institute of Medical Sciences, Deoghar, Jharkhand, India
6 Department of Physical Medicine and Rehabilitation, All India Institute of Medical Sciences, Deoghar, Jharkhand, India
7 Electronics and Communication Engineering, Institute of Engineering and Management, Kolkata, India

* **Correspondence:** Shreya Sharma: Email: shreya1166rv@gmail.com; Tel: +918791622340; Himel Mondal: Email: himelmkcg@gmail.com; Tel: +91983029311.

Supplementary

Table 1. The detailed keywords for the search.

Database	Search Keywords	Filters Applied	Total Results Retrieved
**PubMed**	(“stroke”[tiab] OR “stroke rehabilitation”[tiab]) AND (“repetitive transcranial magnetic stimulation”[tiab] OR “rTMS”[tiab]) AND (“supplementary motor area”[tiab] OR “SMA”[tiab])	English, Human studies, Peer-reviewed, Title/Abstract	19
**Web of Science**	(“stroke”[tiab] OR “stroke rehabilitation”[tiab]) AND (“repetitive transcranial magnetic stimulation”[tiab] OR “rTMS”[tiab]) AND (“supplementary motor area”[tiab] OR “SMA”[tiab])	English, Peer-reviewed, Title/Abstract	61
**Google Scholar**	(“stroke”OR “stroke rehabilitation”) AND (“repetitive transcranial magnetic stimulation” OR “rTMS”) AND (“supplementary motor area” OR “SMA”)	First 10 pages, manually screened, English only	100

AIMS Press

© 2026 the Author(s), licensee AIMS Press. This is an open access article distributed under the terms of the Creative Commons Attribution License (https://creativecommons.org/licenses/by/4.0)
